# Physical Exercise in Major Depression: Reducing the Mortality Gap While Improving Clinical Outcomes

**DOI:** 10.3389/fpsyt.2018.00762

**Published:** 2019-01-10

**Authors:** Martino Belvederi Murri, Panteleimon Ekkekakis, Marco Magagnoli, Domenico Zampogna, Simone Cattedra, Laura Capobianco, Gianluca Serafini, Pietro Calcagno, Stamatula Zanetidou, Mario Amore

**Affiliations:** ^1^Section of Psychiatry, Department of Neuroscience, Ophthalmology, Genetics and Infant-Maternal Science, University of Genoa, Genoa, Italy; ^2^IRCCS Ospedale Policlinico San Martino, Genova, Italy; ^3^Department of Psychological Medicine, King's College London, London, United Kingdom; ^4^Department of Kinesiology, Iowa State University, Ames, IA, United States; ^5^Department of Mental Health, Consultation Liaison Psychiatry Service, Bologna, Italy

**Keywords:** depression, mortality, exercise, physical activity, efficacy, cardiovascular disease

## Abstract

Major depression shortens life while the effectiveness of frontline treatments remains modest. Exercise has been shown to be effective both in reducing mortality and in treating symptoms of major depression, but it is still underutilized in clinical practice, possibly due to prevalent misperceptions. For instance, a common misperception is that exercise is beneficial for depression mostly because of its positive effects on the body (“from the neck down”), whereas its effectiveness in treating core features of depression (“from the neck up”) is underappreciated. Other long-held misperceptions are that patients suffering from depression will not engage in exercise even if physicians prescribe it, and that only vigorous exercise is effective. Lastly, a false assumption is that exercise may be more harmful than beneficial in old age, and therefore should only be recommended to younger patients. This narrative review summarizes relevant literature to address the aforementioned misperceptions and to provide practical recommendations for prescribing exercise to individuals with major depression.

## Introduction

Depression exerts an enormous impact on different domains of individual functioning, as well as physical health (1, 2). Physical exercise is increasingly recognized as an effective intervention to improve these outcomes.

Patients with major depression seldom receive adequate treatment. When they do, there is a high likelihood they remain depressed or relapse after first-line treatment (3, 4). Whereas, a substantial proportion of patients go on to receive intensive pharmacological care (5, 6).

Besides mental health outcomes, recent studies cast great concern on the physical health of depressed individuals. Depression is, in fact, accompanied by behavioral and biological features that are deleterious for physical health, particularly in the cardiovascular system (7). Moreover, when depression arises as a consequence of pre-existing physical problems, it may amplify disability, anticipate recurrences, and increase disease-related mortality (8, 9). Recently it was estimated that individuals with major depression die, on average, about 10 years earlier than those who are not depressed, even when excluding deaths by suicide (10–12).

There is wide agreement that current research and clinical efforts to address these issues are arguably not proportional to their gravity. There is an urgent need to develop and implement novel treatments that are effective to treat symptoms of depression and, at the same time, are beneficial for physical health (13). One such intervention is physical exercise, which is increasingly recognized as both an antidepressant agent (14) and a potent tool to delay mortality (15). The aim of this perspective article is to provide a concise update on the effectiveness of exercise for depression and cardiovascular mortality reduction. A specific section is dedicated to treatment of elderly patients, in consideration of their increasing demographic relevance (2). English-language reviews and meta-analyses published in the last 10 years were considered, identified with the following search string in the Pubmed database: (exercis^*^[ti] OR “physical activity”[ti]) AND depress^*^[ti] AND (review^*^[pt] OR review^*^[tiab]).

## Depression is Associated With a Shorter Lifespan

Even if a direct causal role is still debated (16), depression could increase mortality through several mechanisms (10). First, it negatively impacts lifestyle choices. Individuals with depression tend to be sedentary (17, 18) and less physically fit than their non-depressed counterparts (19). Moreover, they exhibit higher rates of cigarette smoking (20–22), consume more alcohol (23), adopt low-quality dietary regimens (24), and become overweight (25, 26). Of note, some of these associations seem underlined by bi-directional causal links.

Second, depression is accompanied by dysregulation of several homeostatic systems (27). Depressed individuals commonly display dysregulation of the hypothalamic–pituitary–adrenocortical (HPA) axis (28–30), immune (31, 32), and autonomic nervous system (33), as well as metabolic imbalances (34).

Third, depression can raise mortality risk by increasing the incidence of physical illnesses or worsening the outcomes of existing ones. For instance, the presence of clinically significant depression has been found to increase the incidence and mortality of cardiovascular diseases (35, 36), as well as the mortality due to diabetes (37) and stroke (38). This phenomenon could stem, among other reasons, from placing additional stress on disorder-specific pathophysiologic mechanisms, but may also reflect poor adherence to medications or problematic health behaviors (39–41). In this regard, Table 1 reports an overview of the relationship between depression, cardiovascular risk factors, and mortality.

**Table 1:** Literature examining the relationship between depression, cardiovascular risk factors, cardiovascular mortality, and physical exercise in adults.

**Cardiovascular risk factor**	**Association between depression and risk factor**	**Effect of exercise on risk factor among non-depressed populations**
Obesity—overweight	Depression had a 37% increased risk of becoming obese (RR: 1.37, 95%CI: 1.17–1.48); risk was highest for young and middle aged women. Nineteen prospective studies (26, 42)	Exercise was effective to reduce body weight (although less effective than hypocaloric diet) and visceral adipose tissue (more effective than hypocaloric diet). 117 trials (43)
Type 2 Diabetes	Depression was associated with an increased risk of having T2DM (RR: 1.49; 95%CI: 1.29–1.72). Ten studies, only one prospective (44)	Exercise improved Hb1AC levels and insulin resistance. 27 trials (45)
Unbalanced diet	Two out of three studies supported an association between depression and unbalanced diet. Three studies, all cross sectional (24)	na
Blood metabolic parameters	Depression was associated with a higher prevalence of Metabolic Syndrome (OR: 1.54, 95% CI 1.21–1.97), hyperglycemia (OR: 1.33, 95%CI: 1.03–1.73), hypertriglyceridemia (OR: 1.17, 95% CI 1.04–1.30). Eighteen studies, all cross-sectional (34). Depression was associated with lower serum LDL levels (mean difference: 3.15 mg/dL, 95%CI: 6.05–0.24). Eighteen cohort studies (46)	Exercise lowered fasting insulin, HOMA-IR, and Hb1AC levels. TG and APOA1 levels, increased HDL levels; trend-level effects for reductions of LDL and fasting glucose. 160 RCTs (47)
Hypertension	Depression was associated with an increased risk of incident hypertension (RR: 1.42, 95% CI: 1.09–1.86). Nine prospective studies (48)	Exercise reduced blood pressure. The magnitude of the effect changed according to exercise type and was greater for hypertensive subjects. 93 RCTs (49)
Inflammation	Depression was associated with abnormal levels of peripheral cytokines and chemokines compared to HCs. IL-6, TNF-a, IL-10, sIL-2R, CCL-2, IL-13, IL-18, IL-12, and sTNFR2 were significantly elevated, IFN-gamma was slightly reduced. Eighty-two case-control studies (50)	Exercise reduced IL6 and CRP levels in T2DM. Fourteen RCTs (51). Similar results in CAD. Twenty-six trials (52). Possible effect enhancing immune competence and delaying the aging of the immune system (53)
Autonomic dysfunction	Untreated depression was associated with reduced Heart Rate Variability (g: −0.349, 95%CI: −0.51 to−0.19). Twenty-nine case-control studies (54)	Exercise increased HRV in 9 out of 15 trials on T2DM (55). Exercise improved HRV in CAD. Sixteen RCTs (56). Exercise improved HRV in HF. 19 trials (57)
Alcohol use	Depression was associated with increased risk of Alcohol Use Disorders (aOR: 2.09, 95%CI: 1.29–3.38). Seven studies, two of which prospective (23)	Exercise did not reduce daily alcohol consumption or AUDIT total scores. 21 trials (58)
Cigarette smoking	Among adolescents, depression increased the risk of beginning smoking (RR: 1.41, 95% CI: 1.21–1.63). Twelve prospective studies (20). Depressed smokers had lower odds of short-term (OR: 0.83, 95%CI: 0.72–0.95) and long-term abstinence (OR: 0.81, 95%CI: 0.67–0.97). Forty-two clinical trials on smoking cessation (59)	No effect of exercise on smoking cessation. 19 RCTs (60)
Adherence to medications	Depression was associated with an increased likelihood of non-adherence to medications (OR: 1.76, 95%CI: 1.33–2.57). Thirty-one U.S. based cross-sectional studies on chronic diseases (61)	na
Physical inactivity/sedentary behavior	Depression was associated with less time spent for total Physical Activity (SMD: −0.25, 95%CI: −0.03–0.15), higher levels of Sedentary Behavior (SMD: 0.09, 95%CI: 0.01–0.18) and lower likelihood to meet physical activity levels recommended by guidelines (OR: −1.50, 95%CI: −1.10 to −2.10). Twenty-four cross sectional studies (17). A recent large study confirmed the association between mental health and physical activity levels (62)	Exercise interventions yielded uncertain and/or small effects increasing subsequent physical activity (63–65)
**Cardiovascular mortality**	**Association between depression and mortality**	**Effect of exercise on mortality among non-depressed populations**
Coronary heart disease	Depression was associated with an increased risk of myocardial infarction-related death (HR: 1.31, 95%CI: 1.09–1.57) and coronary death (HR: 1.36; 95%CI: 1.14–1.63). Nineteen prospective studies (66). Quality of evidences appraised as “highly suggestive” (16)	Exercise reduced mortality in coronary heart disease (OR: 0.89, 95%Credible Interval: 0.76–1.04) with no difference in magnitude from ACEi, beta-blockers, ARBs and diuretics. Thirty-four RCTs (15). Exercise-based Cardiac Rehabilitation reduced cardiovascular, but not overall mortality (RR: 0.74, 95%CI 0.64–0.86). 27 RCTs (67)
Arrhythmias related mortality	Depression was associated with an increased risk of Sudden Cardiac Death (HR: 1.62; 95%CI: 1.37–1.92), ventricular arrhythmias (HR: 1.47; 95%CI: 1.23–1.76) recurrence of Atrial Fibrillation (HR: 1.88; 95%CI: 1.54–2.30). Seventeen studies, of which 15 prospective (36)	No clear effect of exercise on mortality in Atrial Fibrillation (RR: 1.00; 95%CI: 0.06–15.78). 6 RCTs (68)
Mortality in Heart Failure	Depression was associated with an increased risk of all-cause mortality (HR: 1.20; 95%CI: 1.10–1.31). Increased risk was driven by studies on participants older than 65. 14 prospective studies (35). Quality of evidences was appraised as “highly suggestive” (16)	Exercise reduced mortality in heart failure (OR: 0.79; 95%Credible Interval: 0.59–1.00) to a greater extent than ACEi, beta-blockers, ARBs, but less than diuretics. 18 RCTs (15)
Mortality after Cardiac Surgery	Perioperative depression was associated with an increased risk of early (RR: 1.44; 95%CI: 1.01–2.05) and late postoperative mortality (RR: 1.44; 95%CI: 1.24–1.67). Sixteen prospective studies (69)	Insufficient evidence to establish a significant effect of exercise on mortality after heart valve surgery. 2 RCTs (70)
Overall mortality	Depression was associated with an increased risk of mortality relative to non-depressed participants (RR: 1.52, 95%CI: 1.45–1.59). Excess mortality risk was of similar magnitude in patients from the community vs. those with specific diseases. Two hundred and ninety-three prospective studies (10). Quality of evidence was however appraised as inadequate to support a direct causal association (16, 71)	The network meta-analysis estimated that exercise can reduce mortality to a similar extent to medications among individuals with coronary heart disease, stroke, heart failure, and diabetes. 305 RCTs (15).

*This table summarizes recent literature on: (a) the relationship between depression, cardiovascular risk factors and mortality due to cardiovascular diseases; (b) the effectiveness of exercise modifying such risk factors and mortality. The latest reviews for each topic were identified through multiple searches of the Pubmed database. Quantitative reviews or meta-reviews were preferred over qualitative or narrative ones. The number and type of primary studies is specified (cross-sectional vs. longitudinal; RCTs vs. controlled trials)*.

*Na, not available; RCTs, Randomized Controlled Trials; T2DM, Type 2 Diabetes Mellitus, CAD, Coronary Artery Disease; HF, Heart Failure; ACEi, Angiotensin Converting Enzyme Inhibitors; ARBs, Angiotensin II Receptor Blockers; OR, Odds Ratio; RR, Relative Risk; HR, Hazard Ratio; SMD, Standardized Mean Difference; CI, Confidence Intervals*.

## Exercise is Effective for the Physical Health of Depressed Patients

Physical activity and exercise have a wide range of beneficial effects (72) that involve both “body” and “mind.” Bearing in mind this is an artifactual and anachronistic convention, here we provide an overview of exercise effects on the body “from the neck down” that could be relevant to individuals with depression. Table 1 reports recent literature addressing this issue.

Together with dietary caloric restriction, exercise is the main component of interventions that are effective at reducing and managing weight (73–75). The positive effect of exercise is likely mediated by enhanced regulation of appetite hormones (76) and by increased metabolic rate (47, 77, 78). Moreover, exercise improves sleep quality and duration (79).

Exercise also causes beneficial adaptations in homeostatic systems involved in the response to stress, including the HPA axis (80–82). Moreover, it dampens inflammatory processes while delaying the aging of the immune system (51–53). Exercise also improves the autonomic visceral control by restoring sympathovagal balance (57, 83, 84) Finally, it improves cardiorespiratory fitness both in healthy individuals (47) and individuals with depression (85).

While the formal acknowledgment of the salutary effects of exercise in the medical sciences has been a lengthy process, regular exercise is now recognized as an important lifestyle behavior that can ameliorate the negative impact of chronic diseases (86). Overall, it is estimated that exercise can reduce mortality to a similar extent as medications in individuals with coronary heart disease, stroke, heart failure, and diabetes (15). It would be urgent to verify if such findings can be translated to depressed subjects.

Among the many salutary effects of exercise, arguably the least researched—and probably the most controversial—are its effects on other lifestyle and health behaviors. Both the number of randomized controlled trials and the methodological quality of the trials in this area are rising. While concepts and methods continue to evolve, some early results related to smoking cessation and reducing problem drinking among individuals with mental health disorders show promise (87–89). However, at this stage, systematic reviews of the evidence on the effectiveness of exercise in promoting abstinence from smoking (60) or alcohol (58) indicate no beneficial effect. On the other hand, the effect of exercise on reducing the use of illegal substances is significant (90). In addition, whether a structured exercise intervention can reduce sedentary behavior or encourage engagement in subsequent physical activity remains hotly debated (64).

## Exercise is Effective Against Symptoms of Major Depression

Physical exercise has been shown to be an effective treatment for major depression in adults 14, 91 in several randomized controlled trials comparing it to a wide range of other treatments, including usual care, psychological interventions, and antidepressant medications 14, 92. Although there have been contrarian meta-analytic findings [e.g., 93], closer inspection of methodological details reveals a pattern of debatable choices (91).

Exercise interventions consisting of three sessions per week for 12–24 weeks typically result in a medium to large reduction in the severity of depression, measured by symptom rating scales (91). Moreover, exercise interventions have been found to result in 22% higher likelihood of remission from depression than treatment as usual (93), the latter in turn being associated with the remission of about a third of patients (3, 4). Generally, exercise is well-tolerated and leads to about 18% of dropout rates (94). Based on the available data, the efficacy of exercise seems greater if it is aerobic, delivered in groups, and supervised by an instructor (95). Although there are relatively few head-to-head comparisons and duration of treatment may be different, the efficacy of exercise may be comparable in terms of magnitude to that of psychotherapies (3, 94–97) or antidepressant medications (98).

Some authors claim the psychological effects of exercise largely depend on “placebo,” or “non-specific” psychosocial effects, such as attention by staff (99, 100). Consistently, exercise is still listed among “alternative and complementary” therapies in some guidelines [e.g., (101)]. Skepticism has been fueled, among other reasons, by difficulties to demonstrate a clear dose-response relationship, such as would be expected in drug trials. Recent studies, however, have started to detect significant associations between the intensity and length of exercise interventions, and their antidepressant efficacy (102, 103); of note, such relationship is likely to follow non-linear patterns (104). Another long-held belief among clinicians is that exercise does only ameliorate non-specific somatic symptoms, such as sleep and appetite changes. Whereas, extant results suggest that exercise indeed reduces core symptoms of depression, such as depressed mood, anhedonia, and suicidal ideation (105, 106). On the other hand, studies examining the effects of exercise interventions on cognitive function among individuals with depression [e.g., 107] at present do not indicate substantial benefits (108–110).

Exercise may be effective improving several biomarkers that have been implicated in depression (e.g., impaired neuroplasticity, autonomic, and immune imbalances). However, at present, evidence derived from non-depressed individuals still needs to be replicated among clinical populations (111). Nevertheless, recent trials have begun to show efficacy in treating patients with severe mood disorders (112–114) and individuals with treatment-resistant depression, either alone or as an add-on to medications (115, 116). Lastly, exercise can be effective for individuals who may present concerns about drug treatment, such as women with pregnancy or post-partum depression (117) and adolescents (118, 119).

At present, research is still needed to establish the efficacy of exercise in the long-term course of major depression. Some analyses suggest that the antidepressant effects may diminish beyond the duration of the exercise intervention (92). However, individuals who regularly engage in moderate physical activity maintain reduced risk of incurring depressive episodes (120, 121).

## Effectiveness of Exercise in Late Life Depression

The clinical features and pathophysiology of late-life depression are largely distinct from that encountered among younger adults (122–124). Specifically, depression in late life is associated with a higher prevalence of physical illnesses, greater prevalence of cognitive impairments and inadequate response to antidepressant drugs (125–128). Despite these differences, late-life depression seems to respond to exercise as well as adult depression (129–131). Moreover, among studies appraised in recent meta-analyses, participants receiving exercise did not report any significant side effects. More recently, the SEEDS study showed that exercise was an effective add-on to antidepressant drugs for mild to moderate depression (132). Interestingly, adding exercise to antidepressant drugs primarily affected core symptoms of depression rather than somatic symptoms (133). Moreover, individuals receiving aerobic exercise plus antidepressants displayed greater improvements in cognition and autonomic balance compared to those only receiving antidepressants (134, 135). The intervention was well-received by patients and physicians alike (136).

Despite these promising results, the available evidence remains insufficient to conclude whether exercise can improve cognition in patients with late-life depression (108, 109). At present, studies suggest that exercise may not improve cognition among non-impaired, non-depressed individuals (137), but it may, to some extent, improve cognitive performance among individuals diagnosed with cognitive impairment (irrespective of depression), dementia, or physical diseases (138–141).

## How Should Exercise be Prescribed to Individuals With Depression?

Depression is usually treated by primary care physicians, psychiatrists, and psychologists. Exercise interventions can be delivered by professionals with a variety of disciplinary backgrounds, including group exercise leaders, personal trainers, clinical exercise physiologists, wellness specialists, and physical therapists. Given the challenging cognitive and affective features of depression, it is recommended that exercise for individuals with depression should be delivered by professionals with specific experience in mental health care (142). In other words, a well-integrated, collaborative approach is essential.

A collaborative approach begins with physicians willing to introduce the idea of exercise as a possible treatment options to individuals expressing depression complaints. However, proposals to introduce exercise to the armamentarium of interventions for the treatment of depression are often met with skepticism by physicians due to various perceived barriers (143, 144). These barriers may stem, at least in part, from high-profile reviews and treatment recommendations that downplay the relevant evidence. A recent review, for example, characterized any benefits of exercise, even against non-active control interventions, as merely “modest,” alleged that “high-quality clinical studies investigating the effect of exercise for treating depression among older patients are lacking,” and raised doubt about whether older individuals with depression would be “willing to participate actively in an exercise program” (145). A counterpoint is that, to a large extent, such statements reflect a limited or outdated assessment of the evidence (146, 147). While the evidence base continues to evolve, there are already several randomized controlled trials with positive results that satisfy the standard criteria for high methodological quality (91). Furthermore, provided that proper therapeutic alliances are established within a stepped-care collaborative framework (136), many individuals with subthreshold, mild, and moderate depressive symptoms will opt for exercise and will demonstrate satisfactory adherence.

Several groups have published recommendations for developing exercise prescriptions for individuals with depression, based on both empirical evidence and clinical experience (148–152). While we endorse these recommendations, we should note that the optimal exercise prescription for the treatment of depression remains unknown, insofar as the relation between the “dose” of exercise (i.e., intensity, frequency, session duration) and the therapeutic response remains understudied. Therefore, any prescription recommendations at the present stage are essentially derived from general exercise prescription guidelines, which were developed primarily for the improvement and maintenance of physical fitness and cardiometabolic health (153). Therefore, we wish to highlight an emerging trend in exercise prescription, which may be especially relevant to the treatment of depression, namely affect-based exercise prescription (154). This method expands the traditional focus of exercise prescriptions from the dual goal of maximizing fitness gains while minimizing risk to a model that also aims to ensure that participants consistently derive pleasant affective experiences. The inclusion of pleasure as a central consideration is intended to enhance what is often the Achilles' heel of lifestyle or behavior-change interventions, namely adherence. In a typical affect-based prescription, the exercise participant is shown a simple rating scale (e.g., one ranging from +5: “I feel very good” to −5: “I feel very bad”) and is instructed to self-regulate his or her exercise intensity and duration to maintain a rating of +3 or higher.

Individuals with depression can experience exercise as pleasant and affect-enhancing (155–157). Among non-depressed adults, affective responses to a bout of exercise have been found in correlational studies to be associated with the amount of physical activity individuals choose to do (158), while experimental manipulations resulting in improved affective responses have been shown to increase the amount of physical activity performed over a subsequent period of 6 months (159). Early evidence among individuals with depression indicates that affective responses to a bout of exercise may predict treatment response (160, 161). While randomized controlled trials investigating the efficacy, effectiveness, and cost-effectiveness of affect-based exercise prescriptions for the treatment of depression are not yet available, this method seems to hold promise for clinical application due to its simplicity, making it appealing to physicians who lack specialized training in exercise and to healthcare organizations concerned about implementation costs.

## Conclusions

The premature mortality of individuals with depression is an alarming public health concern, which is exacerbated by the present inability to offer satisfactory treatments. Physical exercise represents an underutilized intervention that may uniquely address both concerns at the same time. First, exercise offers numerous physical benefits, which can counteract several mechanisms postulated to increase mortality risk in depression. Second, if prescribed and delivered correctly, exercise can be as effective as other first-line treatments, while being mostly free of adverse side-effects.

While there is a need of pragmatic trials to evaluate the long-term effects of exercise and its cost-effectiveness, clinicians in the mental health sector should acknowledge this ancient, yet new treatment option and should start to use it to the benefit of patients.

## Author Contributions

MB and PE conceived and drafted the work. MM, DZ, SC, PC, LC, GS, SZ, and MA contributed to revising it critically and approving the content.

### Conflict of Interest Statement

The authors declare that the research was conducted in the absence of any commercial or financial relationships that could be construed as a potential conflict of interest.

## References

[1] Kessler RC. The costs of depression. Psychiatr Clin North Am. (2012) 35:1–14. 10.1016/j.psc.2011.11.005 22370487 PMC3292769

[2] McCall WV Kintziger KW. Late life depression: a global problem with few resources. Psychiatr Clin North Am. (2013) 36:475–81. 10.1016/j.psc.2013.07.001 24229651 PMC3830423

[3] Cuijpers P Karyotaki E Weitz E Andersson G Hollon SD Van Straten A. The effects of psychotherapies for major depression in adults on remission, recovery and improvement: a meta-analysis. J Affect Disord. (2014) 159:118–26. 10.1016/j.jad.2014.02.026 24679399

[4] Kolovos S van Tulder MW Cuijpers P Prigent A Chevreul K Riper H. The effect of treatment as usual on major depressive disorder: a meta-analysis. J Affect Disord. (2017) 210:72–81. 10.1016/j.jad.2016.12.013 28013125

[5] Ijaz S Davies P Williams CJ Kessler D Lewis G Wiles N Psychological therapies for treatment-resistant depression in adults. Cochrane Database Syst Rev. (2018) 2018:CD010558 10.1002/14651858.CD010558.pub2 PMC6494651 29761488

[6] Shen MD Piven J. Brain and behavior development in autism from birth through infancy. Dialogues Clin Neurosci. (2017) 19:325–33. 10.1007/978-3-642-40308-8_2 29398928 PMC5789210

[7] Goldstein BI Carnethon MR Matthews KA McIntyre RS Miller GE Raghuveer G. Major depressive disorder and bipolar disorder predispose youth to accelerated atherosclerosis and early cardiovascular disease: a scientific statement from the american heart association. Circulation (2015) 132:965–86. 10.1161/CIR.0000000000000229 26260736

[8] Gleason OC Pierce AM Walker AE Warnock JK. The two-way relationship between medical illness and late-life depression. Psychiatr Clin North Am. (2013) 36:533–44. 10.1016/j.psc.2013.08.003 24229655

[9] Shaffer JA Edmondson D Chaplin WF Schwartz JE Shimbo D Burg MM. Directionality of the relationship between depressive symptom dimensions and c-reactive protein in patients with acute coronary syndromes. Psychosom Med. (2011) 73:370–7. 10.1097/PSY.0b013e31821deafd 21636659 PMC3110525

[10] Cuijpers P Vogelzangs N Twisk J Kleiboer A Li J Penninx BW. Comprehensive meta-analysis of excess mortality in depression in the general community versus patients with specific illnesses. Am J Psychiatry (2014) 171:453–62. 10.1176/appi.ajp.2013.13030325 24434956

[11] Laursen TM Musliner KL Benros ME Vestergaard M Munk-Olsen T. Mortality and life expectancy in persons with severe unipolar depression. J Affect Disord. (2016) 193:203–7. 10.1016/j.jad.2015.12.067 26773921

[12] Walker ER McGee RE Druss BG Reisinger E McGee RE Druss BG. Mortality in mental disorders and global disease burden implications. a systematic review and meta- analysis JAMA Psychiatry (2015) 72:334–41. 10.1001/jamapsychiatry.2014.2502.Mortality 25671328 PMC4461039

[13] Sampogna G Fiorillo A Luciano M Del Vecchio V Steardo L Pocai B. A randomized controlled trial on the efficacy of a psychosocial behavioral intervention to improve the lifestyle of patients with severe mental disorders: study protocol. Front Psychiatry (2018) 9:235. 10.3389/fpsyt.2018.00235 29930520 PMC6001842

[14] Schuch FB Vancampfort D Richards J Rosenbaum S Ward PB Stubbs B. Exercise as a treatment for depression: a meta-analysis adjusting for publication bias. J Psychiatr Res. (2016) 77:42–51. 10.1016/j.jpsychires.2016.02.023 26978184

[15] Naci H Ioannidis JPA. Comparative effectiveness of exercise and drug interventions on mortality outcomes: metaepidemiological study. Br J Sports Med. (2015) 49:1414–22. 10.1136/bjsports2015f5577rep 26476429 PMC4680125

[16] Machado MO Veronese N Sanches M Stubbs B Koyanagi A Thompson T. The association of depression and all-cause and cause-specific mortality: an umbrella review of systematic reviews and meta-analyses. BMC Med. (2018) 16:1–13. 10.1186/s12916-018-1101-z 30025524 PMC6053830

[17] Schuch F Vancampfort D Firth J Rosenbaum S Ward P Reichert T. Physical activity and sedentary behavior in people with major depressive disorder: a systematic review and meta-analysis. J Affect Disord. (2017) 210:139–50. 10.1016/j.jad.2016.10.050 28033521

[18] Vancampfort D Firth J Schuch FB Rosenbaum S Mugisha J Hallgren M. Sedentary behavior and physical activity levels in people with schizophrenia, bipolar disorder and major depressive disorder: a global systematic review and meta-analysis. World Psychiatry (2017) 16:308–15. 10.1002/wps.20458 28941119 PMC5608847

[19] Papasavvas T Bonow RO Alhashemi M Micklewright D. Depression symptom severity and cardiorespiratory fitness in healthy and depressed adults: a systematic review and meta-analysis. Sport. Med. (2016) 46:219–30. 10.1007/s40279-015-0409-5 26446894

[20] Chaiton MO Cohen JE O'Loughlin J Rehm J. A systematic review of longitudinal studies on the association between depression and smoking in adolescents. BMC Public Health (2009) 9:1–11. 10.1186/1471-2458-9-356 19772635 PMC2758872

[21] Lê Cook B Wayne GF Kafali EN Liu Z Shu C Flores M Trends in smoking among adults with mental illness and association between mental health treatment and smoking cessation. JAMA J Am Med Assoc. (2014) 311:172–82. 10.1001/jama.2013.284985 PMC5555156 24399556

[22] Luger TM Suls J Vander Weg MW. How robust is the association between smoking and depression in adults? A meta-analysis using linear mixed-effects models. Addict Behav. (2014) 39:1418–29. 10.1016/j.addbeh.2014.05.011 24935795

[23] Boden JM Fergusson DM. Alcohol and depression. Addiction (2011) 106:906–14. 10.1111/j.1360-0443.2010.03351.x 21382111

[24] Quirk SE Williams LJ O'Neil A Pasco JA Jacka FN Housden S. The association between diet quality, dietary patterns and depression in adults: a systematic review. BMC Psychiatry (2013) 13:175. 10.1186/1471-244X-13-175 23802679 PMC3706241

[25] Luppino FS de Wit LM Bouvy PF Stijnen T Cuijpers P Penninx BWJH. Overweight, obesity, and depression. Arch Gen Psychiatry (2010) 67:220–9. 10.1001/archgenpsychiatry.2010.2 20194822

[26] Mannan M Mamun A Doi S Clavarino A. Prospective associations between depression and obesity for adolescent males and females- a systematic review and meta-analysis of longitudinal studies. PLoS ONE (2016) 11:e0157240. 10.1371/journal.pone.0157240 27285386 PMC4902254

[27] McEwen BS. Neurobiological and systemic effects of chronic stress. Chronic Stress (2017) 1:10.1177/2470547017692328. 10.1177/2470547017692328 28856337 PMC5573220

[28] Belvederi Murri M Pariante C Mondelli V Masotti M Atti AR Mellacqua Z HPA axis and aging in depression: systematic review and meta-analysis. Psychoneuroendocrinology (2014) 41:46–62. 10.1016/j.psyneuen.2013.12.004 24495607

[29] Gold PW. The organization of the stress system and its dysregulation in depressive illness. Mol Psychiatry (2015) 20:32–47. 10.1038/mp.2014.163 25486982

[30] Stetler C Miller GE. Depression and hypothalamic-pituitary-adrenal activation: a quantitative summary of four decades of research. Psychosom Med. (2011) 73:114–26. 10.1097/PSY.0b013e31820ad12b 21257974

[31] Alexopoulos GS Morimoto SS. The inflammation hypothesis in geriatric depression. Int J Geriatr Psychiatry (2011) 26:1109–18. 10.1002/gps.2672 21370276 PMC3160498

[32] Kiecolt-Glaser JK Derry HM Fagundes CP. Inflammation: depression fans the flames and feasts on the heat. Am J Psychiatry (2015) 172:1075–91. 10.1176/appi.ajp.2015.15020152 26357876 PMC6511978

[33] Sgoifo A Carnevali L Pico Alfonso MDLA Amore M. Autonomic dysfunction and heart rate variability in depression. Stress (2015) 18:343–52. 10.3109/10253890.2015.1045868 26004818

[34] Vancampfort D Correll CU Wampers M Sienaert P Mitchell AJ De Herdt A. Metabolic syndrome and metabolic abnormalities in patients with major depressive disorder: a meta-analysis of prevalences and moderating variables. Psychol Med. (2014) 44:2017–28. 10.1017/S0033291713002778 24262678

[35] Gathright EC Goldstein CM Josephson RA Hughes JW. Depression increases the risk of mortality in patients with heart failure: a meta-analysis. J Psychosom Res. (2017) 94:82–9. 10.1016/j.jpsychores.2017.01.010 28183407 PMC5370194

[36] Shi S Liu T Liang J Hu D Yang B. Depression and risk of sudden cardiac death and arrhythmias: a meta-analysis. Psychosom Med. (2017) 79:153–61. 10.1097/PSY.0000000000000382 27627224

[37] Park M Katon WJ Wolf FM. Depression and risk of mortality in individuals with diabetes: a meta-analysis and systematic review. Gen Hosp Psychiatry (2013) 35:217–25. 10.1016/j.genhosppsych.2013.01.006 23415577 PMC3644308

[38] Pan A Sun Q Oi O Km R Fb H. Depression and risk of stroke morbidity and mortality : a meta-analysis and systematic review. JAMA (2011) 306:2011–2. 10.1001/jama.2011.1282.Depression 21934057 PMC3242806

[39] Goldstein CM Gathright EC Garcia S. Relationship between depression and medication adherence in cardiovascular disease: the perfect challenge for the integrated care team. Patient Prefer Adherence (2017) 11:547–59. 10.2147/PPA.S127277 28352161 PMC5359120

[40] Jonkman NH Westland H Groenwold RHH Ågren S Atienza F Blue L. Do self-management interventions work in patients with heart failure? Circulation (2016) 133:1189–98. 10.1161/CIRCULATIONAHA.115.018006 26873943 PMC5180429

[41] Mausbach BT Schwab RB Irwin SA. Depression as a predictor of adherence to adjuvant endocrine therapy (AET) in women with breast cancer: a systematic review and meta-analysis. Breast Cancer Res Treat. (2015) 152:239–46. 10.1007/s10549-015-3471-7 26077640 PMC4861253

[42] Solmi M Köhler CA Stubbs B Koyanagi A Bortolato B Monaco F. Environmental risk factors and nonpharmacological and nonsurgical interventions for obesity: an umbrella review of meta-analyses of cohort studies and randomized controlled trials. Eur J Clin Invest. (2018) 48: 12982. 10.1111/eci.12982 29923186

[43] Verheggen RJHM Maessen MFH Green DJ Hermus ARMM Hopman MTE Thijssen DHT. A systematic review and meta-analysis on the effects of exercise training versus hypocaloric diet: distinct effects on body weight and visceral adipose tissue. Obes Rev. (2016) 17:664–90. 10.1111/obr.12406 27213481

[44] Vancampfort D Mitchell AJ De Hert M Sienaert P Probst M Buys R. Type 2 diabetes in patients with major depressive disorder: a meta-analysis of prevalence estimates and predictors. Depress Anxiety (2015) 32:763–73. 10.1002/da.22387 26114259

[45] Grace A Chan E Giallauria F Graham PL Smart NA. Clinical outcomes and glycaemic responses to different aerobic exercise training intensities in type II diabetes: a systematic review and meta-analysis. Cardiovasc Diabetol. (2017) 16:37. 10.1186/s12933-017-0518-6 28292300 PMC5351065

[46] Persons JE Fiedorowicz JG. Depression and serum low-density lipoprotein: a systematic review and meta-analysis. J Affect Disord. (2016) 206:55–67. 10.1016/j.jad.2016.07.033 27466743 PMC6201299

[47] Lin X Zhang X Guo J Roberts CK McKenzie S Wu WC. Effects of exercise training on cardiorespiratory fitness and biomarkers of cardiometabolic health: a systematic review and meta-analysis of randomized controlled trials. J Am Heart Assoc. (2015) 4:e002014. 10.1161/JAHA.115.002014 26116691 PMC4608087

[48] Meng L Chen D Yang Y Zheng Y Hui R. Depression increases the risk of hypertension incidence: a meta-analysis of prospective cohort studies. J Hypertens. (2012) 30:842–51. 10.1097/HJH.0b013e32835080b7 22343537

[49] Cornelissen VA Smart NA. Exercise training for blood pressure: a systematic review and meta-analysis. J Am Heart Assoc. (2013) 2:e004473. 10.1161/JAHA.112.004473 23525435 PMC3603230

[50] Köhler CA Freitas TH Maes M de Andrade NQ Liu CS Fernandes BS. Peripheral cytokine and chemokine alterations in depression: a meta-analysis of 82 studies. Acta Psychiatr Scand. (2017) 135:373–87. 10.1111/acps.12698 28122130

[51] Hayashino Y Jackson JL Hirata T Fukumori N Nakamura F Fukuhara S. Effects of exercise on C-reactive protein, inflammatory cytokine and adipokine in patients with type 2 diabetes: a meta-analysis of randomized controlled trials. Metabolism (2014) 63:431–40. 10.1016/j.metabol.2013.08.018 24355625

[52] Swardfager W Herrmann N Cornish S Mazereeuw G Marzolini S Sham L. Exercise intervention and inflammatory markers in coronary artery disease: a meta-analysis. Am Heart J. (2012) 163:666–76. 10.1016/j.ahj.2011.12.017 22520533

[53] Campbell JP Turner JE. Debunking the myth of exercise-induced immune suppression: redefining the impact of exercise on immunological health across the lifespan. Front Immunol. (2018) 9:648. 10.3389/fimmu.2018.00648 29713319 PMC5911985

[54] Alvares GA Quintana DS Hickie IB Guastella AJ. Autonomic nervous system dysfunction in psychiatric disorders and the impact of psychotropic medications: a systematic review and meta-analysis. J Psychiatry Neurosci. (2016) 41:89–104. 10.1503/jpn.140217 26447819 PMC4764485

[55] Villafaina S Collado-Mateo D Fuentes JP Merellano-Navarro E Gusi N. Physical exercise improves heart rate variability in patients with type 2 diabetes: a systematic review. Curr Diab Rep. (2017) 17:110. 10.1007/s11892-017-0941-9 28942507

[56] Nolan RP Jong P Barry-Bianchi SM Tanaka TH Floras JS. Effects of drug, biobehavioral and exercise therapies on heart rate variability in coronary artery disease: a systematic review. Eur J Cardiovasc Prev Rehabil. (2008) 15:386–96. 10.1097/HJR.0b013e3283030a97 18677161

[57] Pearson MJ Smart NA. Exercise therapy and autonomic function in heart failure patients: a systematic review and meta-analysis. Heart Fail Rev. (2018) 23:91–108. 10.1007/s10741-017-9662-z 29185161

[58] Hallgren M Vancampfort D Giesen ES Lundin A Stubbs B. Exercise as treatment for alcohol use disorders: systematic review and meta-analysis. Br J Sports Med. (2017) 51:1058–64. 10.1136/bjsports-2016-096814 28087569

[59] Hitsman B Papandonatos GD McChargue DE Demott A Herrera MJ Spring B. Past major depression and smoking cessation outcome: a systematic review and meta-analysis update. Addiction (2013) 108:294–306. 10.1111/add.12009 23072580 PMC3593055

[60] Klinsophon T Thaveeratitham P Sitthipornvorakul E Janwantanakul P. Effect of exercise type on smoking cessation: a meta-analysis of randomized controlled trials. BMC Res Notes (2017) 10:442. 10.1186/s13104-017-2762-y 28874175 PMC5585974

[61] Grenard JL Munjas BA Adams JL Suttorp M Maglione M McGlynn EA. Depression and medication adherence in the treatment of chronic diseases in the United States: a meta-analysis. J Gen Intern Med. (2011) 26:1175–82. 10.1007/s11606-011-1704-y 21533823 PMC3181287

[62] Chekroud SR Gueorguieva R Zheutlin AB Paulus M Krumholz HM Krystal JH. Association between physical exercise and mental health in 1·2 million individuals in the USA between 2011 and 2015: a cross-sectional study. Lancet Psychiatry (2018) 5:739–46. 10.1016/S2215-0366(18)30227-X 30099000

[63] de Vries NM van Ravensberg CD Hobbelen JSM Olde Rikkert MGM Staal JB Nijhuis-van der Sanden MWG. Effects of physical exercise therapy on mobility, physical functioning, physical activity and quality of life in community-dwelling older adults with impaired mobility, physical disability and/or multi-morbidity: a meta-analysis. Ageing Res Rev. (2012) 11:136–49. 10.1016/j.arr.2011.11.002 22101330

[64] Melanson EL. The effect of exercise on non-exercise physical activity and sedentary behavior in adults. Obes Rev. (2017) 18:40–9. 10.1111/obr.12507 28164451 PMC5388457

[65] Ruotsalainen H Kyngäs H Tammelin T Kääriäinen M. Systematic review of physical activity and exercise interventions on body mass indices, subsequent physical activity and psychological symptoms in overweight and obese adolescents. J Adv Nurs. (2015) 71:2461–77. 10.1111/jan.12696 26031309

[66] Wu Q Kling JM. Depression and the risk of myocardial infarction and coronary death. Med (2016) 95:e2815. 10.1097/MD.0000000000002815 26871852 PMC4753948

[67] Anderson L Thompson DR Oldridge N Zwisler A-D Rees K Martin N Exercise-based cardiac rehabilitation for coronary heart disease. Cochrane Database Syst. Rev. (2016) CD001800. 10.1002/14651858.CD001800.pub3 PMC6491180 26730878

[68] Risom SS Zwisler AD Johansen PP Sibilitz KL Lindschou J Gluud C. Exercise-based cardiac rehabilitation for adults with atrial fibrillation. Cochrane Database Syst. Rev. (2017) 2:CD011197. 10.1002/14651858.CD011197.pub2 28181684 PMC6464537

[69] Takagi H Ando T Umemoto T. Perioperative depression or anxiety and postoperative mortality in cardiac surgery: a systematic review and meta-analysis. Heart Vessels (2017) 32:1458–68. 10.1007/s00380-017-1022-3 28702898

[70] Sibilitz KL Berg SK Tang LH Risom SS Gluud C Lindschou J. Exercise-based cardiac rehabilitation for adults after heart valve surgery. Cochrane Database Syst Rev. (2013) 2013:CD010876. 10.1002/14651858.CD010876 26998683

[71] Miloyan B Fried E. A reassessment of the relationship between depression and all-cause mortality in 3,604,005 participants from 293 studies. World Psychiatry (2017) 16:219–20. 10.1002/wps.20439 28498573 PMC5428195

[72] Ekkekakis P Cook DB Craft LL Culos-reed SN Etnier JL Ginis KAM Routledge Handbook of Physical Activity and Mental Health. London: Routledge (2013).

[73] Ekkekakis P Vazou S Bixby WR Georgiadis E. The mysterious case of the public health guideline that is (almost) entirely ignored: call for a research agenda on the causes of the extreme avoidance of physical activity in obesity. Obes Rev. (2016) 17:313–29. 10.1111/obr.12369 26806460

[74] Fock KM Khoo J. Diet and exercise in management of obesity and overweight. J Gastroenterol Hepatol. (2013) 28:59–63. 10.1111/jgh.12407 24251706

[75] Shaw K Gennat H O'Rourke P Del Mar C Exercise for overweight or obesity. Cochrane Database Syst Rev. (2006). 10.1002/14651858.CD003817.pub3 PMC9017288 17054187

[76] Schubert MM Sabapathy S Leveritt M Desbrow B. Acute exercise and hormones related to appetite regulation: a meta-analysis. Sport Med. (2014) 44:387–403. 10.1007/s40279-013-0120-3 24174308

[77] Marson EC Delevatti RS Prado AKG Netto N Kruel LFM Effects of aerobic, resistance, and combined exercise training on insulin resistance markers in overweight or obese children and adolescents: a systematic review and meta-analysis. Prev Med. (2016) 93:211–8. 10.1016/j.ypmed.2016.10.020 27773709

[78] Umpierre D Ribeiro PAB Kramer CK Leitão CB Zucatti ATN Azevedo MJ. Physical activity advice only or structured exercise training and association with HbA1c levels in type 2 diabetes: a systematic review and meta-analysis. JAMA (2011) 305:1790–9. 10.1001/jama.2011.576 21540423

[79] Kelley GA Kelley KS. Exercise and sleep: a systematic review of previous meta-analyses. J Evid Based Med. (2017) 10:26–36. 10.1111/jebm.12236 28276627 PMC5527334

[80] Chen C Nakagawa S An Y Ito K Kitaichi Y Kusumi I. The exercise-glucocorticoid paradox: how exercise is beneficial to cognition, mood, and the brain while increasing glucocorticoid levels. Front Neuroendocrinol. (2017) 44:83–102. 10.1016/j.yfrne.2016.12.001 27956050

[81] Gerber M Ludyga S Mücke M Colledge F Brand S Pühse U. Low vigorous physical activity is associated with increased adrenocortical reactivity to psychosocial stress in students with high stress perceptions. Psychoneuroendocrinology (2017) 80:104–13. 10.1016/j.psyneuen.2017.03.004 28324699

[82] Klaperski S von Dawans B Heinrichs M Fuchs R. Effects of a 12-week endurance training program on the physiological response to psychosocial stress in men: a randomized controlled trial. J Behav Med. (2014) 37:1118–33. 10.1007/s10865-014-9562-9 24659155

[83] Besnier F Labrunée M Pathak A Pavy-Le Traon A Galès C Sénard JM. Exercise training-induced modification in autonomic nervous system: an update for cardiac patients. Ann Phys Rehabil Med. (2017) 60:27–35. 10.1016/j.rehab.2016.07.002 27542313

[84] Silvani A Calandra-Buonaura G Dampney RAL Cortelli P. Brain-heart interactions: physiology and clinical implications. Philos Trans R Soc A Math Phys Eng Sci. (2016) 374:20150181. 10.1098/rsta.2015.0181 27044998

[85] Stubbs B Rosenbaum S Vancampfort D Ward PB Schuch FB. Exercise improves cardiorespiratory fitness in people with depression: a meta-analysis of randomized control trials. J Affect Disord. (2016) 190:249–53. 10.1016/j.jad.2015.10.010 26523669

[86] Lieberman DE. Is exercise really medicine? An evolutionary perspective. Curr Sports Med Rep. (2015) 14:313–9. 10.1249/JSR.0000000000000168 26166056

[87] Abrantes AM Blevins CE Battle CL Read JP Gordon AL Stein MD. Developing a Fitbit-supported lifestyle physical activity intervention for depressed alcohol dependent women. J Subst Abuse Treat. (2017) 80:88–97. 10.1016/j.jsat.2017.07.006 28755778 PMC5624522

[88] Patten CA Bronars CA Douglas KSV Ussher MH Levine JA Tye SJ. Supervised, vigorous intensity exercise intervention for depressed female smokers: a pilot study. Nicotine Tob Res. (2017) 19:77–86. 10.1093/ntr/ntw208 27613946 PMC5157716

[89] Smits JAJ Zvolensky MJ Davis ML Rosenfield D Marcus BH Church TS. The efficacy of vigorous-intensity exercise as an aid to smoking cessation in adults with high anxiety sensitivity: a randomized controlled trial. Psychosom Med. (2016) 78:354–64. 10.1097/PSY.0000000000000264 26513517 PMC4844851

[90] Wang D Wang Y Wang Y Li R Zhou C. Impact of physical exercise on substance use disorders: a meta-analysis. PLoS ONE (2014) 9:e110728. 10.1371/journal.pone.0110728 25330437 PMC4199732

[91] Ekkekakis P Honey, i shrunk the pooled SMD! Guide to critical appraisal of systematic reviews and meta-analyses using the Cochrane review on exercise for depression as example. Ment Health Phys Act. (2015) 8:21–36. 10.1016/j.mhpa.2014.12.001

[92] Kvam S Kleppe CL Nordhus IH Hovland A. Exercise as a treatment for depression: a meta-analysis. J Affect Disord. (2016) 202:67–86. 10.1016/j.jad.2016.03.063 27253219

[93] Krogh J Hjorthøj C Speyer H Gluud C Nordentoft M Exercise for patients with major depression: a systematic review with meta-analysis and trial sequential analysis. BMJ Open (2017) 7:e014820. 10.1136/bmjopen-2016-014820 PMC5623558 28928174

[94] Stubbs B Vancampfort D Rosenbaum S Ward PB Richards J Soundy A. Dropout from exercise randomized controlled trials among people with depression: a meta-analysis and meta regression. J Affect Disord. (2016) 190:457–66. 10.1016/j.jad.2015.10.019 26551405

[95] Schuch FB Dunn AL Kanitz AC Delevatti RS Fleck MP. Moderators of response in exercise treatment for depression: a systematic review. J Affect Disord. (2016) 195:40–9. 10.1016/j.jad.2016.01.014 26854964

[96] Stubbs B Vancampfort D Rosenbaum S Ward PB Richards J Ussher M. Challenges establishing the efficacy of exercise as an antidepressant treatment: a systematic review and meta-analysis of control group responses in exercise randomised controlled trials. Sport Med. (2016) 46:699–713. 10.1007/s40279-015-0441-5 26707338

[97] Sukhato K Lotrakul M Dellow A Ittasakul P Thakkinstian A Anothaisintawee T. Efficacy of home-based non-pharmacological interventions for treating depression: a systematic review and network meta-analysis of randomised controlled trials. BMJ Open (2017) 7:e014499. 10.1136/bmjopen-2016-014499 28706086 PMC5734422

[98] Cipriani A Furukawa TA Salanti G Chaimani A Atkinson LZ Ogawa Y. Comparative efficacy and acceptability of 21 antidepressant drugs for the acute treatment of adults with major depressive disorder: a systematic review and network meta-analysis. Lancet (2018) 391:1357–66. 10.1016/S0140-6736(17)32802-7 29477251 PMC5889788

[99] Lindheimer JB O'Connor PJ Dishman RK. Quantifying the placebo effect in psychological outcomes of exercise training: a meta-analysis of randomized trials. Sport Med. (2015) 45:693–711. 10.1007/s40279-015-0303-1 25762083

[100] Szabo A. Acute psychological benefits of exercise: reconsideration of the placebo effect. J Ment Heal. (2013) 22:449–55. 10.3109/09638237.2012.734657 23324013

[101] Ravindran AV Balneaves LG Faulkner G Ortiz A McIntosh D Morehouse RL et al Canadian network for mood and anxiety treatments (CANMAT) 2016 clinical guidelines for the management of adults with major depressive disorder. Can J Psychiatry. (2016) 61:576–87. 10.1177/0706743716660290 27486153 PMC4994794

[102] Nebiker L Lichtenstein E Minghetti A Zahner L Gerber M Faude O. Moderating effects of exercise duration and intensity in neuromuscular vs. Endurance exercise interventions for the treatment of depression: a meta-analytical review. Front Psychiatry (2018) 9:305. 10.3389/fpsyt.2018.00305 30072923 PMC6060256

[103] Paolucci EM Loukov D Bowdish DME Heisz JJ. Exercise reduces depression and inflammation but intensity matters. Biol Psychol. (2018) 133:79–84. 10.1016/j.biopsycho.2018.01.015 29408464

[104] Ekkekakis P Parfitt G Petruzzello SJ. The pleasure and displeasure people feel when they exercise at different intensities: decennial update and progress towards a tripartite rationale for exercise intensity prescription. Sport Med. (2011) 41:641–71. 10.2165/11590680-000000000-00000 21780850

[105] Toups M Carmody T Greer T Rethorst C Grannemann B Trivedi MH. Exercise is an effective treatment for positive valence symptoms in major depression. J Affect. (2017) 209:188–94. 10.1016/j.jad.2016.08.058 27936452 PMC6036912

[106] Vancampfort D Hallgren M Firth J Rosenbaum S Schuch FB Mugisha J. Physical activity and suicidal ideation: a systematic review and meta-analysis. J Affect Disord. (2018) 225:438–48. 10.1016/j.jad.2017.08.070 28858658

[107] Ahern E Semkovska M. Cognitive functioning in the first-episode of major depressive disorder: a systematic review and meta-analysis. Neuropsychology (2017) 31:52–72. 10.1037/neu0000319 27732039

[108] Brondino N Rocchetti M Fusar-Poli L Codrons E Correale L Vandoni M. A systematic review of cognitive effects of exercise in depression. Acta Psychiatr Scand. (2017) 135:285–95. 10.1111/acps.12690 28110494

[109] Greer TL Furman JL Trivedi MH. Evaluation of the benefits of exercise on cognition in major depressive disorder. Gen Hosp Psychiatry (2017) 49:19–25. 10.1016/j.genhosppsych.2017.06.002 28690019

[110] Sun M Lanctot K Herrmann N Gallagher D. Exercise for cognitive symptoms in depression: a systematic review of interventional studies. Can J Psychiatry (2017) 63:115–28. 10.1177/0706743717738493 29186973 PMC5788135

[111] Schuch FB Deslandes AC Stubbs B Gosmann NP Silva CTBda Fleck MPde A. Neurobiological effects of exercise on major depressive disorder: a systematic review. Neurosci Biobehav Rev. (2016) 61:1–11. 10.1016/j.neubiorev.2015.11.012 26657969

[112] Legrand FD Neff EM. Efficacy of exercise as an adjunct treatment for clinically depressed inpatients during the initial stages of antidepressant pharmacotherapy: an open randomized controlled trial. J Affect Disord. (2016) 191:139–44. 10.1016/j.jad.2015.11.047 26655860

[113] Minghetti A Faude O Hanssen H Zahner L Gerber M Donath L. Sprint interval training (SIT) substantially reduces depressive symptoms in major depressive disorder (MDD): a randomized controlled trial. Psychiatry Res. (2018) 265:292–7. 10.1016/j.psychres.2018.04.053 29775886

[114] Schuch FB Vasconcelos-Moreno MP Borowsky C Zimmermann AB Rocha NS Fleck MP. Exercise and severe major depression: effect on symptom severity and quality of life at discharge in an inpatient cohort. J Psychiatr Res. (2015) 61:25–32. 10.1016/j.jpsychires.2014.11.005 25439084

[115] Greer TL Trombello JM Rethorst CD Carmody TJ Jha MK Liao A. Improvements in psychosocial functioning and health-related quality of life following exercise augmentation in patients with treatment response but nonremitted major depressive disorder: results from the tread study. Depress Anxiety (2016) 33:870–81. 10.1002/da.22521 27164293 PMC5662022

[116] Mura G Moro MF Patten SB Carta MG. Exercise as an add-on strategy for the treatment of major depressive disorder: a systematic review. CNS Spectr. (2013) 19:496–508. 10.1017/S1092852913000953 24589012

[117] Poyatos-León R García-Hermoso A Sanabria-Martínez G Álvarez-Bueno C Cavero-Redondo I Martínez-Vizcaíno V. Effects of exercise-based interventions on postpartum depression: a meta-analysis of randomized controlled trials. Birth (2017) 44:200–8. 10.1111/birt.12294 28589648

[118] Carter T Morres ID Meade O Callaghan P. The effect of exercise on depressive symptoms in adolescents: a systematic review and meta-analysis. J Am Acad Child Adolesc Psychiatry (2016) 55:580–90. 10.1016/j.jaac.2016.04.016 27343885

[119] Radovic S Gordon MS Melvin GA. Should we recommend exercise to adolescents with depressive symptoms? A meta-analysis. J Paediatr Child Health (2017) 53:214–20. 10.1111/jpc.13426 28070942

[120] Mammen G Faulkner G. Physical activity and the prevention of depression: a systematic review of prospective studies. Am J Prev Med. (2013) 45:649–57. 10.1016/j.amepre.2013.08.001 24139780

[121] Schuch FB Vancampfort D Firth J Rosenbaum S Ward PB Silva ES. Physical activity and incident depression: a meta-analysis of prospective cohort studies. Am J Psychiatry (2018) 175:631–48. 10.1176/appi.ajp.2018.17111194 29690792

[122] Haigh EAP Bogucki OE Sigmon ST Blazer DG. Depression among older adults: a 20-year update on five common myths and misconceptions. Am J Geriatr Psychiatry (2017) 26:107–22. 10.1016/j.jagp.2017.06.011 28735658

[123] Hegeman AJM Kok RM Van Der Mast RC Giltay EJ. Phenomenology of depression in older compared with younger adults: meta-analysis. Br J Psychiatry (2012) 200:275–81. 10.1192/bjp.bp.111.095950 22474233

[124] Naismith SL Norrie LM Mowszowski L Hickie IB. The neurobiology of depression in later-life: clinical, neuropsychological, neuroimaging, and pathophysiological features. Prog Neurobiol. (2012) 98:99–143. 10.1016/j.pneurobio.2012.05.009 22609700

[125] Alexopoulos GS. Depression in the elderly. Lancet (2005) 365:1961–70. 10.1016/S0140-6736(05)66665-2 15936426

[126] Morimoto SS Alexopoulos GS. Cognitive deficits in geriatric depression. Clinical correlates and implications for current and future treatment. Psychiatr Clin North Am. (2013) 36:517–31. 10.1016/j.psc.2013.08.002 24229654 PMC3830452

[127] Taylor WD Aizenstein HJ Alexopoulos GS. The vascular depression hypothesis: mechanisms linking vascular disease with depression. Mol Psychiatry (2013) 18:963–74. 10.1038/mp.2013.20 23439482 PMC3674224

[128] Tedeschini E Levkovitz Y Iovieno N Ameral VE Nelson JC Papakostas GI. Efficacy of antidepressants for late-life depression: a meta-analysis and meta-regression of placebo-controlled randomized trials. J Clin Psychiatry (2011) 72:1660–8. 10.4088/JCP.10r06531 22244025

[129] Bridle C Spanjers K Patel S Atherton NM Lamb SE. Effect of exercise on depression severity in older people: systematic review and meta-analysis of randomised controlled trials. Br J Psychiatry (2012) 201:180–5. 10.1192/bjp.bp.111.095174 22945926

[130] Catalan-Matamoros D Gomez-Conesa A Stubbs B Vancampfort D. Exercise improves depressive symptoms in older adults: an umbrella review of systematic reviews and meta-analyses. Psychiatry Res. (2016) 244:202–9. 10.1016/j.psychres.2016.07.028 27494042

[131] Schuch FB Vancampfort D Rosenbaum S Richards J Ward PB Veronese N. Exercise for depression in older adults: a meta-analysis of randomized controlled trials adjusting for publication bias. Rev Bras Psiquiatr. (2016) 38:247–54. 10.1590/1516-4446-2016-1915 27611903 PMC7194268

[132] Belvederi Murri M Amore M Menchetti M Toni G Neviani F Cerri M Physical exercise for late-life major depression. Br J Psychiatry (2015) 207:235–42. 10.1192/bjp.bp.114.150516 26206864

[133] Murri MB Ekkekakis P Menchetti M Neviani F Trevisani F Tedeschi S. Physical exercise for late-life depression: effects on symptom dimensions and time course. J Affect Disord. (2018) 230:65–70. 10.1016/j.jad.2018.01.004 29407540

[134] Neviani F Murri MB Mussi C Triolo F Toni G Simoncini E. Physical exercise for late life depression: effects on cognition and disability. Int Psychogeriatrics (2017) 29:1105–12. 10.1017/S1041610217000576 28412979

[135] Toni G Belvederi Murri M Piepoli M Zanetidou S Cabassi A Squatrito S. Physical exercise for late-life depression: effects on heart rate variability. Am J Geriatr Psychiatry (2016) 24:989–97. 10.1016/j.jagp.2016.08.005 27660194

[136] Zanetidou S Belvederi Murri M Menchetti M Toni G Asioli F Bagnoli L. Physical exercise for late-life depression: customizing an intervention for primary care. J Am Geriatr Soc. (2017) 65:348–55. 10.1111/jgs.14525 27869986

[137] Kelly ME Loughrey D Lawlor BA Robertson IH Walsh C Brennan S. The impact of exercise on the cognitive functioning of healthy older adults: a systematic review and meta-analysis. Ageing Res Rev. (2014) 16:12–31. 10.1016/j.arr.2014.05.002 24862109

[138] Cai H Li G Hua S Liu Y Chen L. Effect of exercise on cognitive function in chronic disease patients: a meta-analysis and systematic review of randomized controlled trials. Clin Interv Aging (2017) 12:773–83. 10.2147/CIA.S135700 28546744 PMC5436795

[139] Groot C Hooghiemstra AM Raijmakers PGHM van Berckel BNM Scheltens P Scherder EJA. The effect of physical activity on cognitive function in patients with dementia: a meta-analysis of randomized control trials. Ageing Res Rev. (2016) 25:13–23. 10.1016/j.arr.2015.11.005 26607411

[140] Panza GA Taylor BA MacDonald HV Johnson BT Zaleski AL Livingston J. Can exercise improve cognitive symptoms of Alzheimer's disease? J Am Geriatr Soc. (2018) 66:487–95. 10.1111/jgs.15241 29363108

[141] Zheng G Xia R Zhou W Tao J Chen L. Aerobic exercise ameliorates cognitive function in older adults with mild cognitive impairment: a systematic review and meta-analysis of randomised controlled trials. Br J Sports Med. (2016) 50:1443–50. 10.1136/bjsports-2015-095699 27095745

[142] Rosenbaum S Hobson-Powell A Davison K Stanton R Craft LL Duncan M The role of sport, exercise, and physical activity in closing the life expectancy gap for people with mental illness: an international consensus statement by exercise and sports science australia, american college of sports medicine, british association o. Transl J Am Coll Sport Med. (2018) 3:72–3. 10.1249/TJX.0000000000000061

[143] Searle A Calnan M Turner KM Lawlor DA Campbell J Chalder M General Practitioners' beliefs about physical activity for managing depression in primary care. Ment Health Phys Act. (2012) 5:13–9. 10.1016/j.mhpa.2011.11.001

[144] Stanton R Franck C Reaburn P Happell B. A pilot study of the views of general practitioners regarding exercise for the treatment of depression. Perspect Psychiatr Care (2014) 51:253–9. 10.1111/ppc.12088 25307254

[145] Kok RM Reynolds CF III. Management of depression in older adults: a review. JAMA (2017) 317:2114–22. 10.1001/jama.2017.5706 28535241

[146] Ekkekakis P Murri MB Exercise as antidepressant treatment: time for the transition from trials to clinic? Gen Hosp Psychiatry (2017) 49:1–5. 10.1016/j.genhosppsych.2017.11.004 29122144

[147] Netz Y. Is the Comparison between exercise and pharmacologic treatment of depression in the clinical practice guideline of the American college of physicians evidence-based? Front Pharmacol. (2017) 8:257. 10.3389/fphar.2017.00257 28555108 PMC5430071

[148] Baron DA Lasarow S Baron SH Exercise for the treatment of depression. In: Lam LCW Riba M editors. Physical Exercise Interventions for Mental Health. Cambridge: Cambridge University Press (2016). p. 26–40. 10.1017/CBO9781316157565.004

[149] Nyström MBT Neely G Hassmén P Carlbring P. Treating major depression with physical activity: a systematic overview with recommendations. Cogn Behav Ther. (2015) 44:341–52. 10.1080/16506073.2015.1015440 25794191

[150] Rethorst CD Trivedi MH. Evidence-based recommendations for the prescription of exercise for major depressive disorder. J Psychiatr Pract. (2013) 19:204–12. 10.1097/01.pra.0000430504.16952.3e 23653077

[151] Stanton R Happell BM. An exercise prescription primer for people with depression. Issues Ment Health Nurs. (2013) 34:626–30. 10.3109/01612840.2012.758207 23909675

[152] Stanton R Reaburn P. Exercise and the treatment of depression: a review of the exercise program variables. J Sci Med Sport (2014) 17:177–82. 10.1016/j.jsams.2013.03.010 23602562

[153] Garber CE Blissmer B Deschenes MR Franklin BA Lamonte MJ Lee IM. Quantity and quality of exercise for developing and maintaining cardiorespiratory, musculoskeletal, and neuromotor fitness in apparently healthy adults: guidance for prescribing exercise. Med Sci Sports Exerc. (2011) 43:1334–59. 10.1249/MSS.0b013e318213fefb 21694556

[154] Ladwig MA Hartman ME Ekkekakis P Affect-based Exercise Prescription: an Idea Whose Time Has Come? ACSMs Health Fit J. (2017) 21:10–5. 10.1249/FIT.0000000000000332

[155] Mata J Hogan CL Joormann J Waugh CE Gotlib IH. Acute exercise attenuates negative affect following repeated sad mood inductions in persons who have recovered from depression. J Abnorm Psychol. (2013) 122:45–50. 10.1037/a0029881 22985013 PMC11877280

[156] Mata J Thompson RJ Jaeggi SM Buschkuehl M Jonides J Gotlib IH. Walk on the bright side: physical activity and affect in major depressive disorder. J Abnorm Psychol. (2012) 121:297–308. 10.1037/a0023533 21553939 PMC3982878

[157] Weinstein AA Deuster PA Francis JL Beadling C Kop WJ. The role of depression in short-term mood and fatigue responses to acute exercise. Int J Behav Med. (2010) 17:51–7. 10.1007/s12529-009-9046-4 19333764

[158] Rhodes RE Kates A. Can the affective response to exercise predict future motives and physical activity behavior? A systematic review of published evidence. Ann Behav Med. (2015) 49:715–31. 10.1007/s12160-015-9704-5 25921307

[159] Williams DM Dunsiger S Miranda R Gwaltney CJ Emerson JA Monti PM. Recommending self-paced exercise among overweight and obese adults: a randomized pilot study. Ann Behav Med. (2015) 49:280–5. 10.1007/s12160-014-9642-7 25223963 PMC4355095

[160] Rethorst CD South CC Rush AJ Greer TL Trivedi MH. Prediction of treatment outcomes to exercise in patients with nonremitted major depressive disorder. Depress Anxiety (2017) 34:1116–22. 10.1002/da.22670 28672073 PMC5718947

[161] Suterwala AM Rethorst CD Carmody TJ Greer TL Grannemann BD Jha M. Affect following first exercise session as a predictor of treatment response in depression. J Clin Psychiatry (2016) 77:1036–42. 10.4088/JCP.15m10104 27561137 PMC5673095

